# Laterally Positioned Flap Procedure with Augmented or Nonaugmented Palatal Connective Tissue Grafts in the Treatment of Multiple Adjacent Gingival Recessions: A Two-Year Follow-Up Study

**DOI:** 10.3390/ijerph191912208

**Published:** 2022-09-26

**Authors:** Wojciech Bednarz, Jennifer Majer, Justyna Pakuszyńska-Błaszczyk, Marzena Dominiak, Tomasz Gedrange, Agata Zielińska-Pałasz

**Affiliations:** 1 Specialist Outpatient Medical Clinic MEDIDENT, Okulickiego 19 Str., PL 38-300 Gorlice, Poland; 2 Department of Orthodontics, Carl Gustav Carus Campus, Technische Universität Dresden, Fetscherstr. 74, D-01309 Dresden, Germany; 3 Department of Dental Surgery, Medical University in Wroclaw, Krakowska 26 str., PL 50-425 Wroclaw, Poland

**Keywords:** gingival recession, graft, flap, connective tissue, augmentation, palatal mucosa

## Abstract

The most commonly used technique for covering gingival recessions is the coronally advanced flap (CAF) technique due to its high success rate. In clinical situations where there is less keratinized tissue apical to the defect due to unfavorable anatomical conditions, a more advantageous technique for this situation should be considered, specifically the laterally positioned flap (LPF). The aim of this study was to compare the gingival thickness after gingival recession coverage using the laterally positioned flap supported by an augmented and non-augmented connective tissue graft (CTG). Thirty-four patients with 105 gingival recessions of Miller’s class I and/or II were enrolled in this study. The method of choice was the laterally positioned flap. The test group was treated with previously augmented CTG harvested from the palatal mucosa while the control group was treated with a non-augmented CTG. Clinical measurements were recorded at baseline, 6, 12 and 24 months after intervention. Clinical results showed a statistically more significant percentage of average and complete gingival recession coverage in the test group. The LPF in combination with an augmented CTG proves to be an effective alternative to the CAF. Greater improvement in gingival thickness was observed in the LPF with augmented CTG than in non-augmented CTG.

## 1. Introduction

Gingival recession (GR) is defined as the apical shift of the marginal soft tissue below the cemento-enamel junction (CEJ). The resulting exposure of the root surface has been related to several conditions such as dentine hypersensitivity, root caries, cervical abrasion, difficult maintenance of oral hygiene and compromised aesthetics.

The management of gingival recession is based on a comprehensive assessment of the etiological factors and the amount of tissue involvement. The first step in the management of gingival recession should be directed towards eliminating the etiological factors. Subsequently the adequate mucogingival surgical procedure, depending on the dimension of the gingival recession and the local anatomic conditions, needs to be selected [1,2].

Several gingival recession coverage techniques have been introduced over the past years with the aim to restore the gingival recession defect, achieve tissue regeneration and to enhance patients’ aesthetics.

The coronally advanced flap (CAF) is the gold standard technique in case of adequate presence of keratinized tissue apical to the defect. In circumstances where this does not apply due to unfavorable anatomic conditions as for example: very shallow vestibulum, absence of keratinized tissue apical to the defect, presence of gingival cleft, marginal insertion of frenuli; the performance of a laterally positioned flap should be taken into consideration [3].

The laterally positioned flap (LPF) was first introduced by Grupe and Warren in 1956 [4]. The essence of the laterally positioned flap method [4,5,6] is the preparation of the gingiva adjacent to the exposed root surface in such a way that after appropriate tissue mobilization, complete root coverage is achieved. The creation of a full thickness flap initiated by a marginal incision, however, contributed to post-operative complications as in root and alveolar bone exposure at the donor site. Hence, a modification to prevent this complication by conducting the initial incision more apical to the gingival margin was proposed by Grupe [7]. Staffileno [8] on the other hand suggested a lateral flap of partial thickness and Ruben et al. [9] presented a method in which the part of the flap resting on the exposed root surface was of full thickness and its distal part of split thickness. Pfeifer and Heller [10] compared their results after covering gingival recessions with a lateral positioned flap of full or partial thickness and concluded that the full thickness approach yielded significantly better results. Depending on the anatomical condition of the donor site in immediate vicinity to single or multiple gingival recessions, the following covering methods were described: sliding step’s flap, rotating flap, papilla rotating flap and double papilla repositioned flap [7,11].

Furthermore, Caffesse and Espinel [12,13] utilized additionally a free palatal mucosal graft, which was placed into the connective tissue bed at the displaced flap site.

Due to higher predictability and better clinical results, connective tissue grafts (CTG) gained increased popularity in the coverage of gingival recession. For instance, Nelson [14] presented a method using a CTG in combination with a laterally positioned flap and pedicle full-thickness flap to cover multiple gingival recessions. Blanes and Allen [15] proposed a combination of a double papilla repositioned flap, tunnel technique and connective tissue graft to cover two adjacent gingival recessions separated by a high and wide gingival papilla. Harris [16] applied a double repositioned split-thickness pedicle flap to cover a single GR. Zucchelli et al. [3] used for the coverage of single gingival recessions the laterally moved coronal advanced flap technique. The surgical technique of free soft tissue graft and sutured flap were performed in one or two stages [17,18]. Agarwal et al. [19] and Ahmedbeyli et al. [20] applied for the gingival recession coverage, the LPF and acellular dermal matrix allograft technique.

The combination of LPF and autogenous connective tissue graft enhances root coverage stability and reduces the possibility of gingival recession re-appearance in the same sites [21,22].

The palatal masticatory mucosa is the key donor source for obtaining a connective tissue graft for periodontal plastic surgery, due to its high similarity to the keratinized gingival tissue. However, variations in the anatomical morphology as well as minor tissue availability at the prospective palatal donor site can result in reduced graft thickness [23,24].

The harvested tissue-volume from the donor area can severely affect the surgical outcome. Reduced graft thickness can lead to graft shrinkage as well as to a less aesthetic outcome or even graft rejection. In general, the chance for obtaining an acceptable connective tissue graft from a thin palatal mucosa is very unlikely [23].

In order to improve the prognosis of the surgical procedure and to obtain an adequate thickness prior to the gingival recession coverage, the prospective donor site can be augmented with a collagen sponge between the raised palatal flap and bone [24,25].

The aim of the present study was the clinical evaluation and the subsequent comparison of the gingival thickness after gingival recession coverage using a laterally positioned flap with previously augmented and non-augmented CTG.

## 2. Materials and Methods

### 2.1. Study Population

Thirty-four patients with 105 gingival recessions of Miller’s class I and/ or II, with good general health and no contraindication for periodontal surgery, were enrolled in this study and were successfully treated with the LPF technique alongside an augmented or non-augmented CTG to achieve root coverage. The patients’ main complaints were aesthetics and dental hypersensitivity in the affected areas. All patients were examined and treated in the Specialist Outpatient Clinic MEDIDENT in Gorlice, Poland between 2008–2011. The participants have received comprehensive information about the surgical procedure before written consent was obtained for the treatment. The study was conducted in accordance with the recommendations of the Helsinki Declaration of 1975 as revised in 2000 and approved by the Bioethics Committee of Wroclaw Medical University (No. KB—284/1/2008).

**Figure 1:**
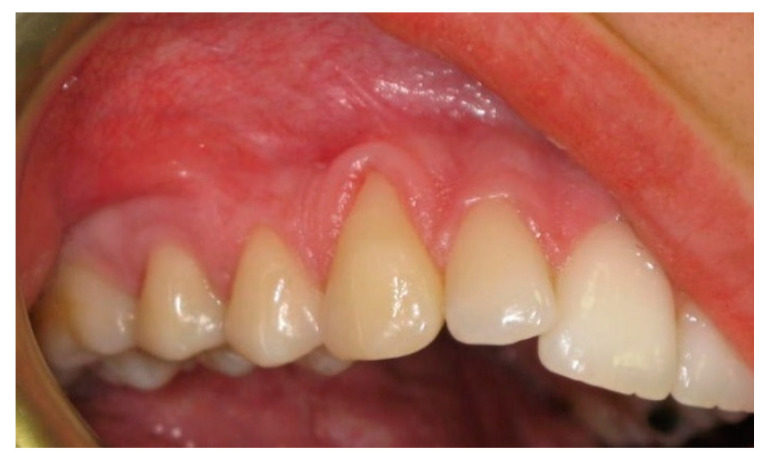
Right canine and first premolar with gingival recession at baseline.

In order to participate in the study, patients had to present at least two gingival recessions of Miller Class I or II (Figure 1) together with the following criteria: (1) between 18 to 60 years of age; (2) full mouth plaque score < 15%; (3) full mouth bleeding score < 10%; (4) clinical attachment level (CAL) ≥ 2 mm (measured mid-buccally); (5) gingival recession depth (GRD) ≥ 2 mm; (6) identifiable cemento-enamel junction (CEJ); (7) absence of previous periodontal surgery at the to be treated sites.

Patients with a history of periodontal disease, presence of systemic diseases that constitute a risk factor for periodontal disease (diabetes, autoimmune diseases, etc.), patients under medication that can influence the structure of the periodontal tissue (e.g., anticonvulsants, calcium channel blockers, immunosuppressants) smoking patients and pregnant or lactating woman were excluded from the study.

All 34 participants received detailed oral hygiene instructions to eliminate the possible etiological factor related to gingival recession. Following the detailed hygiene instruction, all participants adopted to a non-traumatic and accurate oral hygiene throughout the study period.

Depending on the available palatal tissue thickness at the potential donor site for connective tissue collection, the evaluated patients were divided into two groups—the test group and the control group. The measurement points were located on the greatest convexity of the palatal roots at a distance of 4 mm and 8 mm from the gingival margin. Measurements were taken in the area of the canine to the 2nd molar. They were made using the transgingival probing method (description in Section 2.2). In the test group (group-T) the palatal donor site required augmentation with a palatal gingival thickness (PGT) of less than 2.5 mm. In the control group (group-C) there was no need for palatal donor site augmentation with a palatal gingival thickness (PGT) over 2.5 mm.

**Figure 2:**
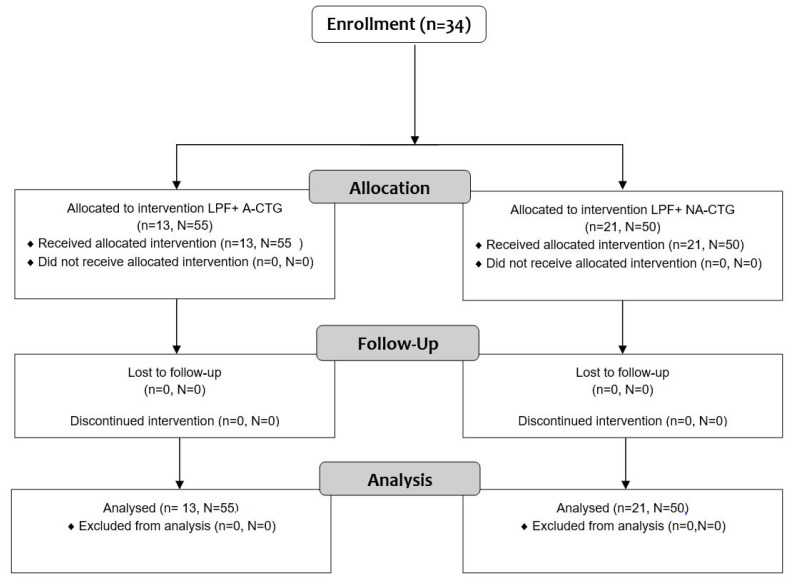
Consort flowchart of the study. LPF, laterally positioned flap; A-CTG, augmented connective tissue graft; NA-CTG, nonaugmented connective tissue graft; n—number of subjects, N—number of gingival recessions.

A flowchart of the study is demonstrated in Figure 2.

### 2.2. Clinical Measurements

Recordings of all clinical parameters were collected by a single investigator (J.P.-B.) in all examinations. The investigator did not perform the surgical procedure and was unaware of the group assignment. Before the study the investigator was calibrated by measuring the extent of multiple adjacent gingival recessions (MAGRs) in three non-study patients repeatedly. A periodontal probe (UNC15, Hu-Friedy, USA) with an accuracy of 1 mm, an endodontic file and a caliper (Iwanson) with an accuracy of 0.1 mm were used for the measurements. The intraexaminer repeatability was accepted with an intraclass correlation coefficient (ICC) of more than 90%.

At baseline, 6 month, 12- and 24-month follow-up appointments, the following parameters were recorded for each treated tooth using an UNC15 periodontal probe: (1) probing depth (PD); (2) clinical attachment level (CAL), (3) gingival recession depth (GRD); (4) gingival recession width (GRW); (5) attached gingiva width (AG); (6) gingival thickness (GT).

The gingival thickness was measured in the presence of attached gingiva in two points located mid-buccally: BGT1 (buccal gingival thickness)—center of keratinized tissue and BGT2 –2 mm apically from the mucogingival junction. In the absence of attached gingiva, GT was recorded mid-buccally, 2 mm apically from the clinical attachment level (BGT3). Measurements at the donor and recipient site were performed under local anesthesia using the transgingival probing method. An endodontic tool with a silicone stop were used and the GT values were read on a caliper (Iwanson) with an accuracy of 0.1 mm.

### 2.3. Surgical Procedures

Prior to the surgical procedure, all patients underwent pre-surgical therapy consisting of scaling and professional tooth cleaning to assure favorable conditions for the surgical intervention.

All mucogingival procedures were performed by the same experienced operator (W.B.).

**Figure 3:**
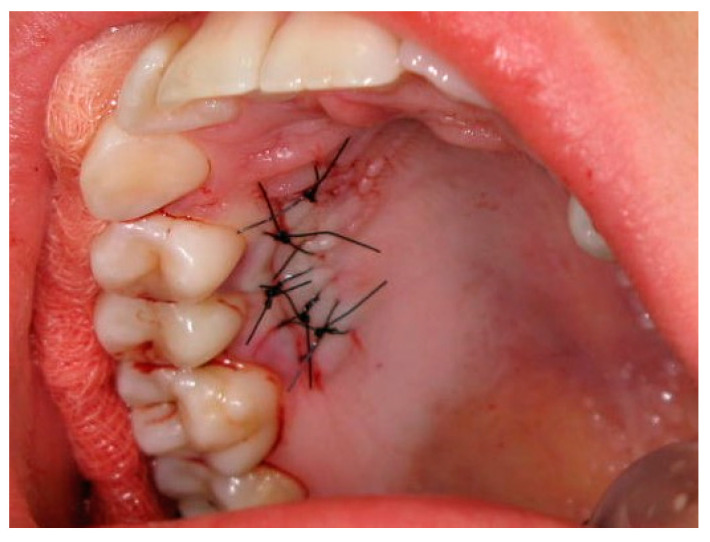
The palatal donor site after harvesting the connective tissue graft.

In both groups, the procedures were performed under local infiltration anesthesia, with a 4% articaine solution and adrenaline at a dilution of 1:100,000 (Septodont^®^, Saint-Maur des Fosses, France). In the control group for the coverage of multiple adjacent gingival recessions, autogenous connective tissue was harvested from the palate via a single incision running parallel to an imaginary tangent line touching the zeniths of the clinical tooth crowns. The resulting palatal wound was secured with a collagen sponge (Biokol^®^ Ravimed Sp. z o.o., Łajski, Poland) and subsequently closed with cross mattress sling sutures with non-resorbable polypropylene monofilament thread (5-0 Dafilon^®^, 16 mm length, 3/8 circle needle, B. Braun Surgical, S.A. Rubi. Spain) (Figure 3).

**Figure 4:**
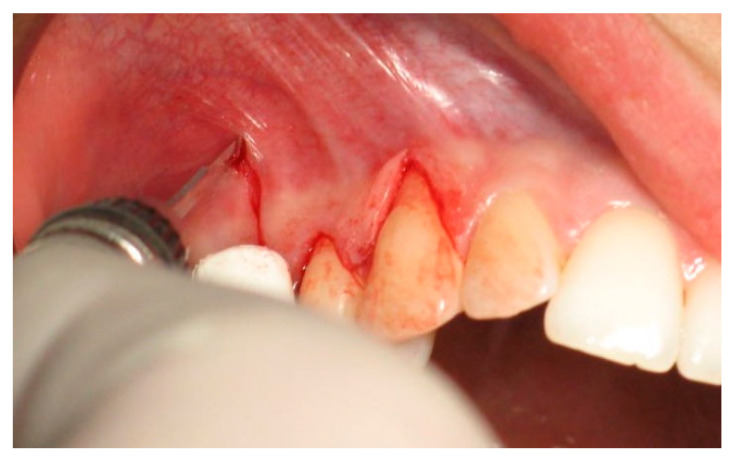
Surgical incisions for flap preparation.

In the test group, 8–10 weeks prior to the main surgical intervention, the augmentation of the palatal mucous membrane at the potential donor site was carried out as formerly described in our previous study [25]. The xenogeneic lyophilized collagen sponge was inserted at the prepared palatal donor site after a split thickness flap was raised at the area from canine to the second molar. Wound closure by cross mattress suturing assured primary surgical wound closure. Thereafter the connective tissue graft was harvested identically as in the control group. In all cases, at the recipient site, the first marginal incision was made at the first exposed root to remove the internal layer of the sulcular epithelium. Next, the two following incisions were performed to obtain one horizontal incision. First, the marginal incision for each remaining tooth to be covered by the procedure and secondly an incision in the interdental space, coronally to the cemento-enamel junction level. Afterwards a vertical incision was carried out, beginning from the base of the interdental papilla of the last tooth with gingival recession, ending below the mucogingival junction (Figure 4).

**Figure 5:**
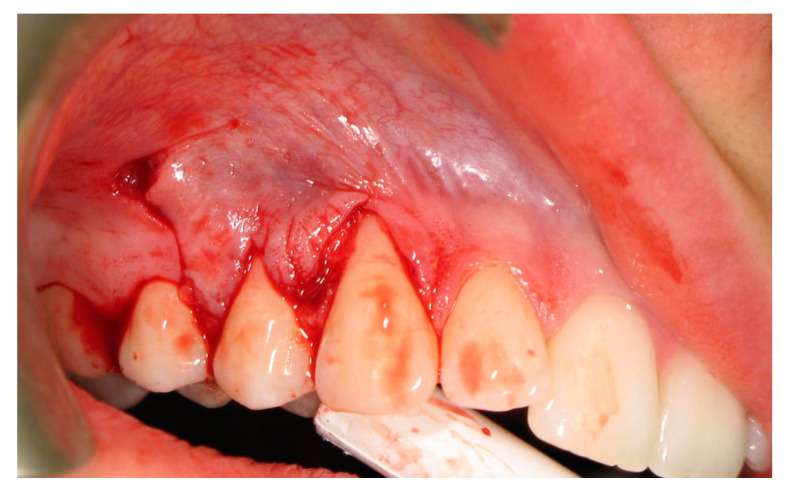
The split-thickness flap.

The partial thickness flap was created supraperiosteal by linking the neighboring teeth spaces with the exposed root surfaces together (Figure 5).

**Figure 6:**
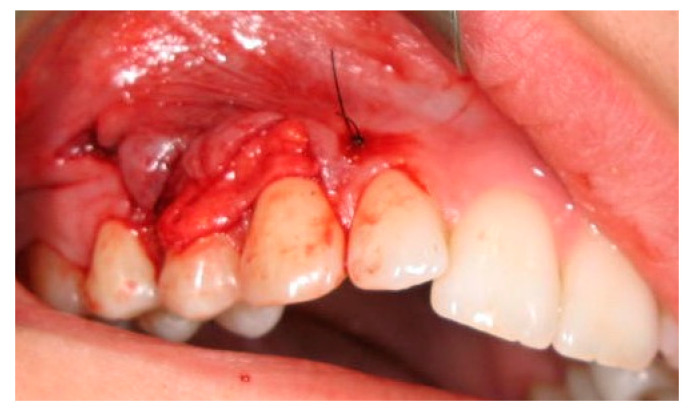
Connective tissue graft placed at the recipient site.

To achieve good mobilization of the split thickness flap, its dimension exceeded vertically the muco-gingival junction. Horizontally it was extended below the papilla of the tooth in close proximity to the operating field, where the supraperiosteal tunnel was created. Afterwards, an autogenous connective tissue graft was inserted in the prepared tunnel as seen in the envelope technique (Figure 6).

**Figure 7:**
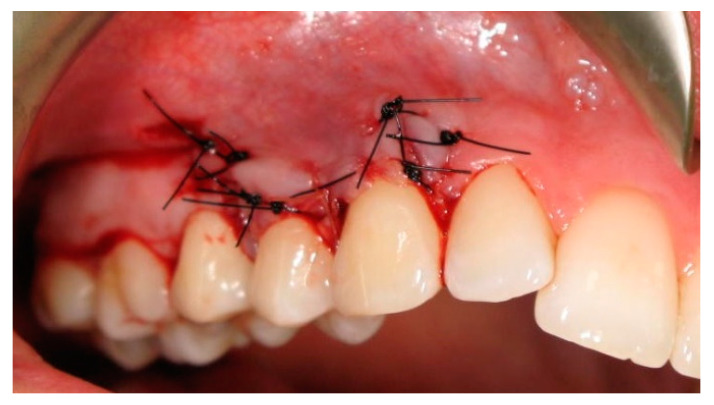
Clinical situation right after the surgical procedure.

The graft was fixed in place using a single sling suture with a non-resorbable polypropylene monofilament thread (6-0 Dafilon^®^, 16 mm length, 3/8 circle needle, B. Braun Surgical, S.A. Rubi. Spain). The remaining graft area was stabilized using a single knotted subperiosteal suture with resorbable material (6-0 Novosyn^®^ Quick, 11 mm length, 3/8 circle needle, B. Braun Surgical, S.A. Rubi. Spain). Subsequently, the split-thickness flap was laterally positioned and sutured to the gingiva adjacent to the gingival recession using mattress sutures (Figure 7).

On each tooth with a presented gingival recession, the sling mattress sutures were placed to advance the flap in a lateral and coronal direction. This allowed to completely cover the exposed root together with the connective tissue graft which was placed previously. Additional sutures connected the flap to the bases of the gingival papillae and at the sites of the vertical releasing incisions. In some surgical wounds partially exposed periosteum and connective tissue were the result of failed primary wound closure.

The following instruments were used during the surgical procedures: round scalpel handle with a microsurgical double-sided scalpel blade SM69 for the recipient site incisions and a BP scalpel blade #15C for the donor site incisions, microsurgical tweezers, tweezers for sewing, Castroviejo needle holder, raspatory for the gingival papilla, gingival scissors, cheek and lip retractors.

To finish off, a surgical dressing (Reso-pac^®^, Hager Werken, Duisburg, Germany) was applied to the donor and recipient sites. Patients were instructed to use cold compresses for one hour after the procedure using dry ice (Top Frost^®^, Akzenta International SA, Switzerland). The surface of the skin in the operated area was advised to be cooled down for 20 min and after a 20 min break cooled again for another 20 min.

### 2.4. Post-Surgical Management

**Figure 8:**
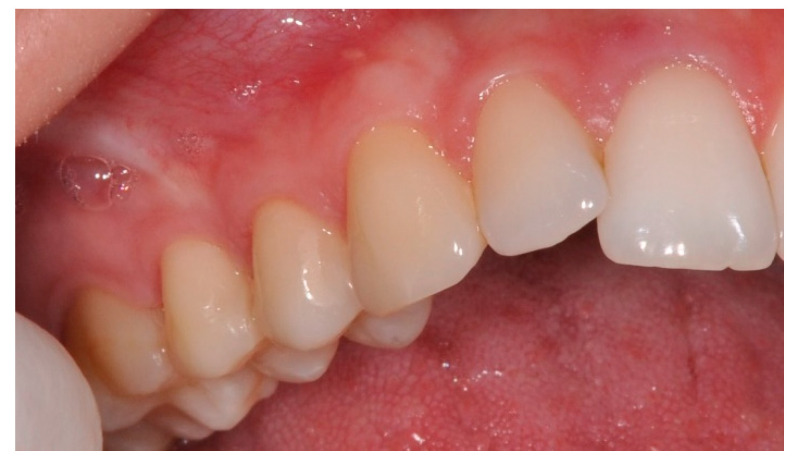
Clinical situation 2 years after surgery with complete root coverage presented.

Postoperative care was carried out following the standard protocol as described in the previous study [25] After the surgical procedure, the patients controlled plaque levels chemically with a 0.1% Chlorhexidine oral mouthwash, 3 times a day. Patients were instructed to discontinue tooth brushing at the surgical site for 7 days and to withhold from dental flossing for 2 months. Subsequently, the patients were guided to use an ultra-soft post-operative toothbrush and 3 weeks after post-treatment the usage of a soft toothbrush was directed. Initially, every 7 days and then every 14 days between the 4th and the 8th week the patients’ plaque was removed professionally. The patients adhered to a liquid diet on the first day, a semi-liquid diet for the next 3 days, followed by a soft diet up to the 14th day. Surgical sutures were removed 14 days after surgery. Figure 8 shows the clinical condition 2 years after covering the gingival recession on tooth 14 and 13 by a laterally positioned flap together with a non-augmented connective tissue graft.

### 2.5. Statistical Analysis

Mean values (x), and standard deviations (SD) of the studied continuous parameters were calculated for all groups. The normality of distribution was tested with the Shapiro–Wilk test and homogeneity of variance was verified using Barlett’s test.

Verification of the hypothesis about the equality of the mean parameters in independent groups was conducted using the ANOVA variance analysis method or, for groups with heterogeneous variance, using the non-parametric U Mann–Whitney’s test. Verification of the hypothesis on the equality of the mean parameters in dependent groups (the baseline values with the 6-, 12- and 24 months postoperative values) was conducted using the Wilcoxon signed rank test. *p* ≤ 0.05 has been established as statistically significant. The statistical analysis was performed with the software program Statistica 13.1.

Percentage of average root coverage (ARC), percentage of complete root coverage (CRC), percentage of clinical attachment level gain (%CAL) and percentage of gingival thickness increase (%GTI) for BGT1, BGT2, BGT3 were calculated for 6, 12 and 24 months post-surgery as follows:

$$\mathrm{ARC}=\frac{\mathrm{GRDbaseline}-\mathrm{GRD}X}{\mathrm{GRD}0}\times 100\%;\;\mathrm{CRC}=\frac{\mathrm{nCRC}}{\mathrm{all}\;\mathrm{GR}}\times 100\%;$$

$$\%\mathrm{CAL}=\frac{\mathrm{CALbaseline}-\mathrm{CAL}X}{\mathrm{CALbaseline}}\times 100\%;\;\%\mathrm{GTI}=\frac{\mathrm{BGT}X-\mathrm{BGTbaseline}}{\mathrm{BGTbaseline}}\times 100\%.$$

*X* = corresponding value 6, 12 or 24 months post-surgery.

## 3. Results

In 34 patients, 47 multiple gingival recession coverage procedures were performed. 105 gingival recessions of Class I and II according to Miller were covered using the LPF and CTG method. 23 cases were treated with a graft harvested from the augmented palatal mucosa, and 24 cases without augmentation. The distribution of treated defects was primarily in the incisor area (45 sites) and least frequently in the molar area (3 sites). There were 50 gingival recessions in the maxilla and 55 in the mandible (Table 1). Table 2 demonstrates the measured clinical parameters at baseline, 6, 12 and 24 months after intervention and Table 3 the descriptive statistics recorded between 6, 12 and 24 months after surgery.

### 3.1. Clinical Measurements for CAL and GRD

Comparing the mean measurements at baseline of CAL and GRD, there were no statistically significant differences between the test and control groups, while the values of postoperative measurements at 6, 12 and 24 months after the procedure showed significant statistical differences between both groups except GRD at 6 months. Moreover, in both groups there was an increase in the postoperative values. In the test group, the baseline value was 4.64 ± 0.97 mm (CAL_baseline_) and 24 months after the procedure was 1.26 ± 0.87 mm (CAL_24_). Similarly, in the control group, the baseline value was 4.43 ± 0.36 mm (CAL_baseline_) and 24 months postoperative was 1.67 ± 0.89 mm (CAL_24_). A statistically significant difference was also visible in the GRD measurements before and after the treatment. Correspondingly, in the test group before the procedure, GRD was 3.05 ± 0.93 mm (GRD_baseline_) and 24 months after the procedure yielded 0.28 ± 0.55 mm (GRD_24_), and in the control group GRD amounted 3.11 ± 1.10 mm (GRD_baseline_), and 0.57± 0.67 mm (GRD_24_), respectively.

### 3.2. Clinical Measurements for GRW, PD and AG

Both the initial PD measurement and the postoperative PD measurements at 6, 12 and 24 months did not show statistical differences between the control group and the test group. In the test group before the treatment PD was 1.56 ± 0.55 mm (PD_baseline_) and after 24 months amounted 1.29 ± 0.45 mm (PD_24_), and in the control group PD was 1.36 ± 0.50 mm (PD_baseline_), and 1.27 ± 0.43 mm (PD_24_), respectively. The mean width of the gingival recession before treatment in the test group was 3.38 ± 0.78 mm (GRW_baseline_), and similarly in the control group was 3.11 ± 0.82 mm (GRW_baseline_), while it decreased significantly 6 months after treatment, amounting to 0.53 ± 0.94 mm, 1.05 ± 1.26 mm, respectively. There was also a statistically significant difference in the baseline AG measurements and 6, 12, 24 months after the procedure. Before the procedure in the test group AG was 0.93 ± 1.30 mm (AG_baseline_) and in the control group it was 1.37 ± 1.37 mm (AG_baseline_) and 2 years postoperative yielded 3.09 ± 1.10 mm and 2.36 ± 1.20 mm, respectively. There were also significant statistical differences when comparing AG between groups 6, 12, 24 months after treatment (Table 2).

### 3.3. Clinical Measurements for BGT1, BGT2, BGT3

The differences in the baseline measurement of BGT1, BGT2, BGT3 in the test (T) and control groups (C) were not statistically significant and were, respectively: BGT1_baseline_ 1.06 ± 0.24 mm (T) and 1.05 ± 0.25 mm (C), BGT_aseline_ 1.04 ± 0.31 mm (T) and 0.97 ± 0.28 mm (C) BGT3_baseline_ 0.93 ± 0.23 mm (T) and 0.95 ± 0.29 mm (C). Postoperative, the gingival thickness values in all three measurement sites increased and were statistically significantly higher in the test group compared to the control group. After 2 years, BGT1_24_ in the test group was 1.64 ± 0.23 mm and in the control group 1.35 ± 0.18 mm, BGT2_24_ was 1.63 ± 0.24 mm (T) and 1.31 ± 0.16 mm (C), BGT3_24_ 1.53 ± 0.24 mm (T) and 1.33 ± 0.29 mm (C), respectively.

### 3.4. Clinical Values for CRC and ARC

After 6 months from the procedure, the percentage of average root coverage in the test group was 92.0 ± 14.3% and in the control group it was 85.7 ± 17.1%. The difference between the groups increased with the passage of time and 2 years after the procedure it was 91.5 ± 16.4%, 83.4 ± 19.0%, respectively, which was statistically significant (Table 3).

Percentage of complete root coverage 6 months postoperative in the test group was 69.1% and 54.0% in the control group and 70.9 and 52.0, respectively 24 months after the procedure. When comparing the two groups, a statistically significant difference is visible, showing a greater percentage of complete gingival recession coverage in the test group.

## 4. Discussion

Recent years have brought an extremely rapid development of surgical techniques in mucogingival surgery. Most of the innovations presented by the authors are based on a combination of different methods, combining flap displacements with the use of autogenous connective tissue or its substitutes [14,20,26]. Some papers describe modified preparations of the recipient sites, yet others present a special technique of surgical suturing [3,27]. Macrosurgical methods are being replaced by microsurgical ones, performed under optical magnification, in which accurate primary closure of the surgical wound using microsurgical instruments, atraumatic needles with appropriate threads, favor rapid, undisturbed vascularization of the graft and healing per primam intentionem [2,28,29]. The most anticipated postoperative situation is for the non-vascular exposed root surface to be covered, in addition to the connective tissue graft, with a mucosal flap of at least 0.8 mm thickness, which would cover the graft completely and the edge of the flap would be situated at least 2 mm above the cemento-enamel junction [30,31]. This results in very high rates of complete gingival recession coverage. According to Ozcelik et al. [31] the size of the exposed root surface area, the gingival thickness and width are the most important parameters in predicting the success of complete gingival recession coverage.

Carnio [32] performed a two-stage procedure in 30 patients for single GR coverage with the LPF method. In the first stage, he extended the gingiva using the modified apically repositioned flap method to perform the elementary procedure after 8 weeks. The authors achieved a statistically significant increase in gingival width for the study group from 2.78 mm to 5.07 mm. After 18 months, the mean depth of gingival recession decreased from 1.86 mm at baseline to 0.57 mm. A very important factor, determining not only the post-operative clinical and aesthetic result, as well as the choice of surgical treatment method, is the thickness of the surrounding soft tissues in immediate vicinity to the tooth with gingival recession (when planning coronally advanced flap procedure) and neighboring teeth (when planning a laterally positioned flap procedure or applying the tunnel technique) [17,25,31].

The transgingival probing method is used most commonly in measuring gingival and palatal mucosal thickness using a needle or endodontic tool with a 3 mm diameter silicone stop [33,34,35,36,37,38,39,40]. At the recipient site, the edge of the stop after puncturing the gingiva should adhere to the gingival margin, resulting in a midbuccal measuring point located 1.5 mm in apical direction. Subsequently, the obtained GT value is read using a caliper. In our study, in areas with a present Miller class I gingival recession, the gingival thickness was measured at 2 points: in the middle of the keratinized tissue and 2 mm apically from the mucogingival junction similar as seen in the study by da Silva et al. [33]. In case of Miller class II gingival recessions, the measurement was taken 2 mm apically from the clinical attachment level [25].

Leong et al. [41] suggested that in thin gingival biotypes namely with GT < 1 mm a CTG should be used and in thick gingival biotypes (≥1 mm) no CTG is needed. Other authors suggested the utilization of a CTG as advisable in recipient sites where soft tissue thickness accounts equal to or less than 0.7 mm and/or there is no keratinized gingiva available [42,43]. Cairo et al. [36] proved a significant improvement in CRC after CAF/CTG procedures, however only in sites where initial gingival thickness was ≤0.8 mm, measured mid-buccally 1.5 mm apical to the gingival margin. This shows great importance in the coverage of multiple gingival recessions. Especially, when reduced harvested graft size prevents it from being placed over all affected teeth.

In the event of soft tissue thickness variations at the recipient site, the connective tissue graft can be suitably extended or fragmented for the placement in areas with the thinnest gingival phenotype.

In a similar study, comparing short- and long-term results in the treatment of multiple gingival recessions Pini Prato et al. [30] likewise confirmed the percentage stability of CRC in the group treated with the CPF/CTG method and a percentage drop in CRC for the CPF alone group in the 5-year observation period.

According to these authors, the utilization of a CTG not only increases keratinized tissue thickness and width but also influences the improvement of clinical results.

Previous studies have also confirmed the influence of the size and quality of the connective tissue graft on the effectiveness of covering multiple gingival recessions [16,23,25]. Augmentation of potential CTG donor sites in case of thin palatal mucosa significantly affects the clinical results achieved in procedures to cover multiple gingival recessions [25].

There are only few current reports in the available literature evaluating the laterally positioned flap technique in covering gingival recessions [3,17,20,22,26,44]. Most commonly, these are case reports describing the coverage of single narrow and high RD or Still man clefts, in conditions of gingival absence, shallow oral vestibule or pulling syndrome [19,22,26,44,45,46]. This technique is also used in soft tissue reconstruction with or without connective tissue grafting after Epulis resection [44,47]. Chambrone et al. [48] evaluated in five patients the soft tissues, 3–4 months after covering single gingival root recessions located on the mesio-buccal root of upper first molars, using the LPF method. Root- and soft tissue of the treatment area were obtained and examined histomorphometrically. The presence of new attachment, gingival crevicular epithelium, long junctional epithelium and new connective tissue attachment was verified.

Furthermore, clinical studies confirm the high efficacy and predictability of the laterally positioned flap method without and with autologous or allogeneic connective tissue augmentation [3,20,44,49].

The laterally positioned flap technique according to Chambrone et al. [50] with recipient site de-epithelialization and the Laterally Moved Coronal Advanced Flap technique (LMCAF) according to Zucchelli et al. [3] for the treatment of single gingival recessions are the most commonly evaluated. In our study, at the gingival papilla site adjacent to the gingival recession, a supraperiosteal tunnel was created for the connective tissue graft placement. Using a similar surgical technique at the recipient site, so called LPF-tunnel technique with the application of a connective tissue graft, Fan et al. [47] covered three single gingival recessions in the maxilla, two of Class III and one of Class II acc. to Miller. After 12 months follow-up, they achieved an average coverage of 78.9%. The GT value increased from 0.83 mm to 1.83 mm, the mean value of width of keratinized tissue (WKT) from 0.83 mm at baseline to 5.50 mm and the mean value of CAL decreased from 6.00 mm at baseline to 1.83 mm, 12 months post-surgery. Zucchelli et al. [3]—using the LMCAF method covered 120 gingival recessions achieving 80% complete root coverage at 12 months follow-up. CAL decreased about 4.4 ± 1.0 mm and WKT gain was 2.2 ± 0.7 mm. If the WKT of the flap was ≥2 mm, the probability of CRC increased 2-fold (87%) compared to the WKT in the flap of 1 mm (42%).

In the study of Zucchelli et al. [49] 50 single gingival recessions of Miller class I and II located on first molars in 50 patients were treated in two groups of 25. The first group was treated with the (LMCAF) while the second was treated with the bilaminar technique (BT), a coronally advanced flap with connective tissue graft. There was more CRC and a greater increase in GT in the BT group and a greater increase in WKT in the LMCAF group after 12 months. The average gingival recession coverage was 88.8 ± 11.2% for the BT with CTG group and 74.2 ± 8.2% for the LMCAF group. Complete root coverage was 48% and 4%, respectively.

Santana et al. [17] in a randomized clinical trial compared the CAF and LPF technique in 36 single gingival Miller class I/II gingival recessions in maxillary incisors, canines and premolars. After 6 months the mean root coverage was 95.5% in the LPF group and 96.6% in the CAF group and complete root coverage was achieved in 83.33% and 88.88%, respectively. The mean WKT values were statistically better in LPF.

Ahmedbeyli et al. [20] used LPF alone and LPF-ADM in 22 patients with single GR where GT was less than 0.8 mm and GRD was greater than 3 mm. After 12 months, ARC was 77.25% in the LPF alone group and 94.8% in the LPF-ADM group. CRC was 45.45% and 72.73%, respectively. The differences were statistically significant, which was also underlined by the KT and GT gain in favor of the procedure using a connective tissue substitute.

Two studies were found evaluating the use of LPF + CTG in covering multiple gingival recessions [14,21].

In Nelsons research [14], 29 gingival recessions were covered in 14 patients. After 3.5 years follow-up, a mean gingival recession coverage of 91% was found. At baseline, in twenty cases RD was 7–10 mm, in six cases RD was 4–6 mm and in three cases RD was 1–3 mm. The complete root coverage was equal 50%, 67% and 100%, respectively.

Ricci et al. [21] used the same technique as Nelson, namely the split-thickness laterally positioned flap together with a connective tissue graft (20 cases) and full-thickness LPF only (20 cases) for coverage of single GR acc. to Miller class I /II. The mean root coverage after 1-year follow-up was 61.9% in the LPF only group and 76.55% in CTG + LPF group. There were no statistically significant differences. The CRC was 44.40% in the CTG + LPF group and 20% in the LPF group. The CRC gain in the LPF group was 3.7 mm and in the CTG + LPF group 2.12 mm. The difference was statistically significant.

In the current study, there were significantly better clinical results in ARC and CRC at 12 months than in the study presented by Ricci et al. [21] and similar ARC at 24 months as in the study by Nelson [14]. In our own study, we evaluated the width of the attached gingiva, which increased at 12 months by 1.01 mm in the control group and by 2.15 mm in the test group. The mean PD values in both evaluated groups did not change significantly during the 2-year observation period.

Only one study assessing and comparing the use of augmented and non-augmented connective tissue in the coverage of multiple adjacent gingival recessions (MAGRs) was found in the available literature [25]. The authors used the same protocol for soft tissue management of palatal mucosa donor sites as in this study. However, the treatment method at the recipient site was a coronally positioned flap. After 2 years of gingival recession coverage with augmented CTG, they obtained a mean gingival recession coverage of 91.7% and a complete root coverage of 67.5%, which is comparable to the results obtained in the current study, and in the non-augmented CTG group 89.9% and 57.7%, respectively, which is slightly higher than our current study (ARC—83.4% and CRC—52%). Similarly, slightly higher %CAL results were obtained compared to the current study, with the greatest difference in the control group at 2 years 71.98% versus 61.5%. The mean increases in GT1, %GT2 and %GT3 were lower compared to the current study, especially in the test group, 2 years post-surgery in areas with Miller class II gingival recessions (%GT3)—34.21% versus 75.0%. In contrast, better widening of attached gingiva was obtained compared to the current study. This is not consistent with the results of other studies, in which keratinized tissue gain is significantly greater in procedures with a coronally positioned flap compared with a laterally positioned flap.

At every observation stage, significant differences were demonstrated between the test and control group in terms of the assessed clinical parameters, with the exception of mean PD and GRW values. These values confirmed the validity for augmenting potential connective tissue donor sites.

In the test group, the lack of statistically significant differences in the assessed clinical parameters between 12 and 24 months of observation confirm the stability of the obtained clinical results. On the other hand, the group using non-augmented CTG showed only statistically significant clinical attachment loss during this period.

It is necessary to extend the observation time over a longer period. Hence, this study is continuing.

The limitation of the study was the lack of randomization due to the qualification into study groups, depending on the need for connective tissue augmentation at the potential palatal donor site.

## 5. Conclusions

As presented in this study, the laterally positioned flap together with the connective tissue graft shows good and predictable clinical results. It proves to be a satisfying treatment option in the coverage of multiple adjacent gingival recessions. Overall, the augmentation procedure of a potential donor site showed greater improvement in gingival thickness, average root coverage and complete root coverage as in comparison to the non-augmented procedure. Therefore, in the case of a thin palatal mucosa at the potential donor site, it is recommended to augment with a lyophilized collagen sponge.

## Figures and Tables

**Table 1:** Patient related records.

	Test Group (LPF + A − CTG)	Control Group (LPFF + NA − CTG)	Total
Number enrolled patients	13 (10 females, 3 males)	21 (14 females, 7 males)	34 24F 10M
Number surgical procedures	23	24	47
Number covered recessions	55	50	105
Number type of recession (Miller)			
*I*	23	29	52
*II*	32	21	53
Number treated teeth per patient			
*Mean value ± SD*	4.23 ± 2.17	2.38 ± 1.60	
*Range*	2–10	1–6	
Number localisation of recessions			
*Incisor*	18	27	45
*Canine*	16	11	27
*Premolar*	19	11	30
*Molar*	2	1	3
*Maxilla*	33	17	50
*Mandible*	22	33	55

LPF, Laterally positioned flap; A-CTG, Augmented connective tissue graft; NA-CTG, Non-augmented connective tissue graft; SD—standard deviation.

**Table 2:** Clinical parameters at baseline, 6, 12 and 24 months after intervention.

Mean Value in mm ± Standard Deviation									
Group/Time		CAL	PD	GRD	GRW	AG	BGT1	BGT2	BGT3
Test group (CAF + A − CTG)	Baseline	4.64 ± 0.97	1.56 ± 0.55	3.05 ± 0.93	3.38 ± 0.78	0.93 ± 1.30	1.06 ± 0.24	1.04 ± 0.31	0.93 ± 0.23
	6 months	1.15 ± 0.78 *^,^**	1.17 ± 0.45 *	0.26 ± 0.47 *	0.53 ± 0.94 *	3.25 ± 1.12 *^,^**	1.64 ± 0.24 *^,^**	1.63 ± 0.22 *^,^**	1.55 ± 0.22 *^,^**
	12 months	1.31 ± 0.92 *^,^**	1.32 ± 0.44 *	0.32 ± 0.62 *^,^**	0.67 ± 1.14 *	3.08 ± 1.14 *^,^**	1.64 ± 0.23 *^,^**	1.63 ± 0.21 *^,^**	1.54 ± 0.21 *^,^**
	24 months	1.26 ± 0.87 *^,^**	1.29 ± 0.45 *	0.28 ± 0.55 *^,^**	0.67 ± 1.14 *	3.09 ± 1.10 *^,^**	1.64 ± 0.23 *^,^**	1.63 ± 0.24 *^,^**	1.53 ± 0.24 *^,^**
Control group (CAF + NA − CTG)	Baseline	4.43 ± 1.36	1.36 ± 0.50	3.11 ± 1.10	3.11 ± 0.82	1.37 ± 1.37	1.05 ± 0.25	0.97 ± 0.28	0.95 ± 0.29
	6 months	1.66 ± 0.89 *^,^**	1.21 ± 0.42 *	0.49 ± 0.60 *	1.05 ± 1.26 *	2.28 ± 1.23 *^,^**	1.37 ± 0.18 *^,^**	1.31 ± 0.16 *^,^**	1.35 ± 0.27 *^,^**
	12 months	1.84 ± 0.89 *^,^**	1.24 ± 0.38 *	0.63 ± 0.74 *^,^**	1.17 ± 1.27 *	2.38 ± 1.12 *^,^**	1.36 ± 0.17 *^,^**	1.31 ± 0.16 *^,^**	1.35 ± 0.30 *^,^**
	24 months	1.67 ± 0.89 *^,^**	1.27 ± 0.43	0.57 ± 0.67 *^,^**	1.13 ± 1.27 *	2.36 ± 1.20 *^,^**	1.35 ± 0.18 *^,^**	1.31 ± 0.16 *^,^**	1.33 ± 0.29 *^,^**

* statistically significant difference in comparison to baseline (Wilcoxon signed-rank test); ** between-groups statistically significant difference (ANOVA; Mann–Whitney *U* test); LPF, Laterally positioned flap; A-CTG, augmented connective tissue graft; NA-CTG, nonaugmented connective tissue graft; CAL, clinical attachment level; PD, probing depth; GRD, gingival recession depth; GRW, gingival recession width; AG, attached gingiva width; BGT_1,2,3_, buccal gingival thickness.

**Table 3:** Descriptive statistics recorded between 6, 12 and 24 months post-surgery.

Percentage % ± Standard Deviation							
Group/Time		ARC	%CAL	%BGT_1_	%BGT_2_	%BGT_3_	CRC
Test group (CAF + A − CTG)	6 months	92.0 ± 14.3	74.9 ± 16.1 **	60.3 ± 32.5 **	65.8 ± 36.2 **	78.0 ± 54.9 **	69.1 **
	12 months	90.6 ± 17.7 **	71.6 ± 18.6 **	60.5 ± 32.9 **	60.5 ± 32.9 **	76.4 ± 52.9 **	70.9 **
	24 months	91.5 ± 16.4 **	72.6 ± 17.6 **	60.5 ± 33.4 **	64.4 ± 34.6 **	75.0 ± 54.3 **	70.9 **
Control group (CAF + NA − CTG)	6 months	85.7 ± 17.1	61.8 ± 15.9 **	31.4 ± 39.3 **	42.6 ± 30.7 **	48.1 ± 31.8 **	54.0 **
	12 months	81.7 ± 20.8 **	57.1 ± 17.8 **	30.2 ± 37.9 **	42.6 ± 30.7 **	47.6 ± 29.2 **	50.0 **
	24 months	83.4 ± 19.0 **	61.5 ± 16.3 **	29.3 ± 36.9 **	42.6 ± 30.7 **	46.0 ± 30.0 **	52.0 **

** between-groups statistically significant difference (ANOVA; Mann–Whitney *U* test) LPF, Laterally positioned flap; A-CTG, augmented connective tissue graft; NA-CTG, nonaugmented connective tissue graft; ARC, percentage of average root coverage; %CAL, percentage of clinical attachment level gain; %BGTI_1,2,__3_, percentage of buccal gingival thickness increase; CRC, percentage of complete root coverage.
